# The Evolving Approach to Breast Cancer: Moving toward De-Escalating Treatment and Personalized Medicine

**DOI:** 10.3390/cancers15133502

**Published:** 2023-07-05

**Authors:** Thaer Khoury

**Affiliations:** Department of Pathology, Roswell Park Cancer Institute, Elm & Carlton Streets, Buffalo, NY 14263, USA; thaer.khoury@roswellpark.org

In recent years, more attention has been directed to personalized medicine in the management and treatment of breast cancer (BC). A new concept has been introduced, “less is more”, meaning that less treatment is better in some instances. With the increase of treatment options comes the risk of over-treatment. In this Special Issue, all ten of the studies published within aim to describe methods of treatment de-escalation in BC, whether through original research or review. These studies can be grouped into three categories: a de-escalating surgical approach for BC in general and pre-neoplastic high-risk lesions in particular; cosmesis and radiation therapy (RT); and chemotherapy (CT)/immunotherapy (IT).

The incidence of pre-neoplastic low-risk ductal lesions, including atypical ductal hyperplasia and ductal carcinoma in situ (DCIS), has increased in recent years. With this increase, the number of operations performed has increased as the standard of care is surgical removal of these lesions. However, the benefit of this approach has been questionable. Therefore, several clinical trials have been initiated to investigate the natural history of low-risk DCIS. These trials might recommend active surveillance for a subset of these patients based on trial outcomes. In their review, Khoury et al. reviewed all these trials in terms of eligibility criteria and found that there was substantial variation between them, predicting difficulty in standardizing a treatment plan. Moreover, they explained the issues with the design of these studies, particularly with the inherited difficulty in differentiating between ADH and DCIS and between intermediate and high-grade DCIS. A real concern was raised regarding the possibility of leaving significant disease without treatment, such as invasive carcinoma or worse metastatic carcinoma to the axillary lymph node (ALN) [1,2]. Nonetheless, these trials are expected to provide some insight into the natural history of low-risk DCIS. They may also provide some treatment recommendations, particularly for those who may not benefit from an aggressive treatment such as surgical excision with or without RT, and with or without hormonal therapy. It is noted that these trials have experienced difficulty in recruiting patients, as the decision of whether to excise or observe is not simple to make [1]. Byng et al. discussed preferences of treatment strategies among women with low-risk DCIS and oncologists. Interestingly, they found that women were more interested in the option of active surveillance and tolerating the risk of developing ipsilateral invasive BC than oncologists. Both patients and oncologists favored less aggressive locoregional treatments, and both demonstrated a strong preference for having a twice-yearly follow-up compared to annual follow-up [3].

On the same subject, Niwińska et al. retrospectively reviewed 737 consecutive DCIS cases with a 15-year follow-up. They reported 66 recurrences (42% DCIS, 58% invasive). Interestingly, they reported that the highest number of recurrences was among the lowest-risk group who were not treated with RT. Although 0.5% of all patients died from DCIS progression, 10.5% who developed invasive carcinoma died. However, most of the deaths were due to a reason other than DCIS, including older age and other diseases, including other cancers. This finding raised the question of whether there is a method of identifying patients who will not benefit from the addition of RT [4]. This question was answered by Angarita et al.’s review, in which the authors mentioned that de-escalating RT could be achieved by decreasing the duration, the extent of the breast to be radiated, and even omitting RT altogether. Potential approaches were discussed, including hypofractionation [5,6] and accelerated partial breast irradiation [7].

An example of de-escalating RT was presented by Ott et al. when they evaluated the risk of radiogenic pneumonitis following external beam accelerated partial breast irradiation (APBI). Patients with early BC were prospectively enrolled in this trial, in which they received 3-6-fold dose reduction compared to whole-breast irradiation (WBI). They found that only 1/170 (0.6%) patients developed radiogenic pneumonitis [8]. It was proven that low-risk patients achieve a similar clinical outcome when they are treated with APBI compared to WBI [9]. Therefore, de-escalating RT by reducing the dose would achieve similar clinical outcomes but with reduced side effects.

De-escalating the surgical approach in BC has evolved over the years from the first type of surgery, radical mastectomy, to selectively excising multiple lesions or even to no surgery at all. Angarita et al. [2] updated these approaches by reviewing clinical trials that compared mastectomy and breast-conserving therapy (BCT). They iterated that BCT with RT is safe and achieves similar results to mastectomy. A de-escalating surgical approach after a positive margin has also evolved, with recent evidence showing no benefit of margins wider than “no ink on tumor” [10,11]. This approach changed the method of reporting the status of the margin in the pathology report. In a neoadjuvant setting with an increased rate of pathologic complete response (pCR), a surrogate variable for good clinical outcomes, the interest in omitting surgery on this subset of patients has increased. However, there are several obstacles, including the lack of a robust method or algorithm that reliably predicts pCR with a very low false negative rate; the fact that pCR is not a guarantee of disease-free survival; and where RT fits in this approach. Furthermore, surveillance strategies will be required, as the false negative rate will never be 0% and some cancers will be missed [2].

In the meantime, patients who undergo surgical resection post-neoadjuvant CT can undergo level II oncoplastic surgery (OPSII) as an alternative option to mastectomy with immediate breast reconstruction (MIBR). Leone et al. presented their data to compare patients who underwent OPSII vs. those who underwent MIBR in terms of local disease-free survival, regional disease-free survival, distant disease-free survival, overall survival, aesthetic results, and quality of life. The selected patients had locally advanced disease with significant residual disease post-neoadjuvant CT. They found similar survival outcomes between the two groups, but patients who were treated with OPSII had a lower loss of breast sensitivity after surgery and better physical well-being of the chest. However, the study is retrospective from a single institution with a relatively small sample size and relatively short follow-up time. Therefore, these results should be interpreted with caution, and a prospective and well-designed study is encouraged [12]. Nonetheless, this de-escalating approach to the appropriate type of surgery for locally advanced disease with significant residual disease post-neoadjuvant CT fits well with the current direction of using less to achieve similar or even better outcomes. Another study evaluated two types of breast reconstructions: implant-based and synthetic TIGR-mesh-based. The patient population included those who had higher risk of BC, including BRCA1, BRCA2, PALB2, or CHEK2 mutation. They found that implant-based breast reconstruction with the use of synthetic mesh is a safe and effective method of breast restoration, associated with low morbidity and good cosmesis. However, the study suffered from some limitations, including short follow-up time and lack of homogeneity in some confounding factors. Therefore, a prospective multicenter study with long-term outcome data is needed to evaluate these results [13].

The approach to ALNs has evolved throughout the years with the introduction of the sentinel lymph node (SLN) technique, and, more recently, the omission of ALN dissection after a diagnosis of positive SLN and the treatment of those patients with whole-breast irradiation [14]. While neoadjuvant CT downstages BC, allowing patients who become node-negative to undergo SLN biopsy to evaluate nodal pathological complete response, there is a need to further de-escalate ALN surgery. Like in the adjuvant setting, in the neoadjuvant setting, there is an opportunity to de-escalate the ALN treatment from ALN dissection to ALN radiation for those who become clinically node-negative (NCT01901094).

There is a very specific clinical presentation in which the patient is diagnosed with DCIS on core biopsy and the subsequent resection shows invasive carcinoma. The clinical question is whether there are specific clinical or pathological variables that could predict a very low risk of residual disease in the axilla, and therefore to suggest omitting the SLN biopsy altogether. Yu et al. presented their experience by recruiting 316 candidate cases. They found two variables correlated with positive SLN: the presence of lymphovascular invasion and tumor size larger than 5 mm. When they applied these two filters, only 2.6% of the patients had positive SLN. This number decreased further to 1.3% when they included only patients treated with a surgery other than BCT [15].

Another pressing clinical issue related to LN metastasis is metachronous isolated supraclavicular nodal metastasis. The supraclavicular fossa triangle is defined by the omohyoid muscle and tendon, the internal jugular vein, and the clavicle and subclavian vein. The area above the triangle is considered the lower cervical node region and the involved LN is staged as M1 [16]. Therefore, surgical intervention is considered contraindicated. Although Chen et al.’s study is not necessarily focused on the theme of treatment de-escalation, it is relevant because they presented a different and more effective treatment plan for patients with this unusual clinical presentation. They found that patients who underwent neck LN dissection had better 5-year overall survival than those who did not have neck dissection. It is important to note, however, that this study is retrospective and suffered from selection bias even though the authors controlled for confounding factors in their statistical analysis [17].

Chemotherapy is another area that needs to be re-evaluated. Fortunately, the number of treatments and clinical trials has substantially increased in the last two decades. The BC type that has been most studied in recent years is triple-negative BC (TNBC), as it does not have targeted therapies such as ER+ or HER2+, and it has the worst clinical outcomes. The new advancements include chemotherapies, immunotherapies, and targeted therapies. However, with these aggressive therapies come significant, sometimes life-threatening side effects. It is also clear that a subgroup of patients with TNBC may experience similar benefits from a less aggressive therapy. Gupta et al. conducted a comprehensive review of completed and ongoing clinical trials. They also suggested ways to maximize benefit with avoiding unnecessary toxicities [18]. Focusing on a specific category, using IT of pembrolizumab in the neoadjuvant setting followed by adjuvant pembrolizumab per KEYNOTE-522 [19], pathologic complete response was achieved in 64.8 % of those who were treated with IT/CT vs. 51.2% in the placebo/CT group. However, immune-related adverse events (irAEs) were observed in 43.6% of patients with IT/CT vs. 21.9% in the placebo/CT group. It is clear that patients who achieved pCR with CT alone (51.2%) were exposed to toxic IT with no added benefit. There is a large body of ongoing research to identify this subset of patients.

In conclusion, there have been real attempts to de-escalate various treatment modalities, including surgery, type of surgery, RT, CT, and IT. It is expected that with the increase of the number of treatment options, more studies are going to emerge to address overtreatment and recommend de-escalation, presenting positives for patient care.
